# Class in contemporary Britain: comparing the Cultural Capital and Social Exclusion (CCSE) project and the Great British Class Survey (GBCS)

**DOI:** 10.1111/1467-954X.12286

**Published:** 2015-06-12

**Authors:** Elizabeth B Silva

**Affiliations:** 1 Open University

**Keywords:** class, cultural capital, social exclusion, CCSE, GBCS, Great Britain

## Abstract

The paper discusses the salience of class in Britain in relation to the experiment of the BBC–academic partnership of the Great British Class Survey (GBCS). It addresses the claimed inauguration of a third phase in class analysis in the UK sparked by the experiment. This is done by considering three main issues. First, the GBCS experiment is situated in the context of various explorations of cultural class analyses, and chiefly in relation to the Cultural Capital and Social Exclusion (CCSE) project (ESRC funded 2003–6). Secondly, the focus is on the influence of the academic turn to big data for the procedures and claims of the project, and some implications of the methodological choices. Thirdly, attention is turned to the deleterious effects of commercial and institutional pressures on the current research culture in which the experiment exists.

## Introduction

It is common to think that class is a serious matter in Britain; as seen in ordinary conversations, in the media and even in academic discussion, it is presumed that class does not have great, or greater, importance elsewhere. This is not true, and it is puzzling. This vision disregards that all capitalist societies are organized around class divisions because these are the basis of elementary workings of capitalist economies. Divisions of social class, based on ranking, hierarchies and inequalities are found nearly everywhere. However, the specific formation and particular relationality of social classes are bound by their cultural milieu, and productive sociological approaches used to identify and analyse class will be affected by, and have to account for, these specific markers.^1^

If a pervasive notion of culture informs academic approaches to class, varied ontologies inform controversies. There is agreement that whether the criteria for class analyses are based on ownership of the means of production, on occupation or on possession of different types of capitals, contextual culture inflects the specific class divisions and their significance for individuals. But the way culture is taken in as an explanatory factor changes the understanding and significance of the matter.

Perhaps it is not that puzzling that the British think their ‘class society’ is such a privileged case. Being the birth place of the industrial revolution, negotiating labour rights for a longer period than most countries, having long-standing aristocratic culture and monarchic power and privilege, housing Marx’s writing of *Capital* at the British Library, may count as good historical reasons for the salience of the concerns with class. Public perceptions of this unduly class-ridden society appear to have a greater resonance in England than in Scotland, Wales or Northern Ireland and concern the legacies of a culture of deference, more akin to status in Weber’s sense (group differentiation on the basis of honour, prestige, or the like) than to class as actual economic inequality (grouped on the basis of shared economic interests).

This imaginary of the unique importance of class in Britain was fed into and fuelled by the experiment of the Great British Class Survey (GBCS) in 2010–11.^2^ The experiment is said to have captured the imagination of the British population and it had considerable international repercussions, in particular in the former colonies of the United States, Australia and New Zealand, where current class divisions are more strongly informed and imagined in reference to (for or against) the British one, for the very reasons of their particular imperial links as capitalist historical formations.

The GBCS is a British Broadcasting Corporation (BBC) project, developed with academic sociologists and used for sociological analysis. The BBC funded both the web survey and the face-to-face survey. The experiment added to other BBC interventions nourishing debates on social mobility with programmes such as ‘Who gets the best jobs’ (2011), Melvin Bragg’s on ‘Class’ (2012) and a different popular format by Paul O’Grady’s ‘Working Britain’ (August 2013).^3^

This experiment appears as a good case to examine academic contentions concerning the subject of social stratification research, the role of academics in constituting significant social events linked to public interest and the impact of research matters.

For this special issue I have been asked to discuss the methods, and relations between, the ‘Cultural Capital and Social Exclusion’ Project (CCSE), the main findings of which were published in *Culture, Class, Distinction* (Bennett *et al*., 2009), and the Great British Class Survey (GBCS). I was reluctant to do this because CCSE is a joint research project and also I may not yet have achieved the degree of reflexivity required for an engagement of this sort. Moreover, as an academic affiliated with the Centre for Research on Socio-Cultural Change (CRESC), I have worked in various roles with nearly everyone in the GBCS team, and I am aware that any robust critique could leave me open to the possible charge of hubris. Nevertheless, being among a uniquely placed group, which had worked on another thorough and substantive study (CCSE), I have taken up the challenge hoping to clarify for me, and to share with others, some of the reservations I have with GBCS regarding the methods of the research and dissemination, as well as the results and claims made for these.

To start, I consider the GBCS project and its broad links with other cultural analyses of class, discussing some discrepancies between GBCS and other versions. I focus more extensively on CCSE. I explore the potential legacies available to GBCS, seeking to understand how the same theoretical approaches generated divergent methodologies and analytical developments, and how these resulted in different findings and conclusions. Secondly, I look at some detail of the (big) data generated by GBCS and some key findings. My attention focuses in particular upon the procedures, the operationalization of concepts (or lack of it) and the relation between the two kinds of survey used in this experiment. The current academic engagement with big data and its institutional (and intellectual) implications are reflected upon in relation to the GBCS case. Thirdly, I address the public reach and impact of GBCS. For this, I attend to the relations between academic and media fields and the potential pitfalls of publicity, to discuss the deleterious effects of commercial and institutional pressures on research culture. To account for these issues I draw on the original paper published in April 2013 (Savage *et al*., 2013), a second paper published online in June 2014 as a response to the academic debate (Savage *et al*., 2014), the introduction to this special issue by Devine and Snee, 2015, and the original 2010 questionnaire used for the BBC survey.

## The project

Three valuable undertakings of the GBCS project can be highlighted:

1. It seeks to offer an *approach* to class analysis different from that of the National Statistics Socio-economic Classification (NS-SEC), which is based on a model of class derived from measures of employment relations (remuneration, career opportunities and time autonomy) attending to relational aspects between categories (see Mills, 2015).
2. It sets out to assess empirically whether classes might be formed as ‘entities’, which straddle particular measures of economic, cultural, and social capital, by *inductively* examining underlying relationships between discrete economic, social and cultural variables (using latent class analysis).
3. It stresses the interplay of *multiple* different factors in the formation of class, such as education, age and location.

While these three aspects relate to theoretical and envisaged empirical assets of the project, the authors make two other important claims in the original paper published in the British Sociological Association (BSA) journal, *Sociology*. One is that their project created the largest survey ever conducted in the UK, and the other that it inaugurated a new phase in class analysis – a third phase.^4^

GBCS researchers claim that a new model of class analysis was needed because of increasing social polarization (between top and bottom) of the measures of inequality, and the fragmentation of the middle layers. To engage with this, they argue it is fundamental to move away from a conventional sociological framing centred on the economic, recognizing that social and cultural processes also generate class divisions. This is the key point about the approach used in this project, and the one upon which I shall concentrate.

I want to centre the discussion of the GBCS project on the claim for the need of a new model, in a new phase, starting in January 2011 with the BBC online survey. The claim for the need for large data, and the legacy of the data set it leaves behind, as well as the perceived ability to ask ‘comprehensive questions’ in a survey are also related to the creation of the new model, and I discuss these in the section that follows. My intention is that this discussion should serve as a way of engaging with reflection about the judgements made throughout the research process. While the focus is on relevant points in the design and the results, it is useful to reflect about some key legacy of the cultural analyses of class upon which GBCS rests.

The central tenet of cultural class analysis is that culture is embedded in economic and social relations, as a central mechanism for generating hierarchies and inequalities. It acknowledges the centrality of lifestyle and taste in social life as a resource in social position. Pierre Bourdieu’s (1984) work has been pivotal in the development of various versions of cultural analyses which dispute the centrality of different assets and the ways they work in the constitution of class. This theme was first developed in the UK before the wide dissemination of Bourdieu’s work in English language. Notable is the study by Willis (1977) *Learning to Labour*. Cultural analyses of class were productively developed by feminist academics before being taken up by mainstream sociology. American sociological production is also relevant. Educational credentials are seen as having a strong role in the strategy of class competition in the explorations by Reay (1998) and Lareau (2003), for example. Moral class boundaries are presented as symbolic differentiation not based on exclusions by Lamont (1992), who also expanded the field into ethno-racial classification. Sayer (2005) finds, on the other hand, that life chances are objectively affected by class, which affects the parameters of value and self-worth. Gender divisions are shown to be directly constitutive of class relations in the work of Bradley (1998), Lawler (2000) and Walkerdine *et al*. (2001). Other powerful intervention about the connections of class and gender is found in the work of Crompton (2000), although Crompton, with Scott, maintains the need to distinguish between some elements of the cultural and the economic, and to attend to this distinction methodologically (Crompton and Scott, 2005). Expanding the focus, social divisions in cultures of sexuality are examined by Moran and Skeggs (2004). Within the turn to culture approaches, class identity has varied salience, no longer emphasized as class-consciousness or unified dispositions, but found in practices of creation of social identity, premised on differentiation from others and on disidentification processes resulting from particular class positions, as investigated by Skeggs (1997) and Savage (2000). Later on, Skeggs (2004) explores the relations between culture and property in the making of class distinctions. These are some key exemplars of cultural class analyses in the UK. There are many more elaborations and nuances in them, which just sample the field. Clearly we have achieved a rich understanding of cultural constructions of class, although no consensual view exists about what is cultural class analysis, or about the proper appropriations of the Bourdieusian model (see Silva and Warde, 2010). It is also important to note that researchers in the GBCS team are not equally committed to Bourdieu’s approach.

Within this varied field the most extensive recent empirical explorations of cultural class analysis in the UK are those of the Cultural Capital and Social Exclusion (CCSE) project and the GBCS. I turn to some comparisons between them.

The questionnaire used for the BBC web survey, and the face-to-face one, is a simplified (and in a few points bettered) version of the questionnaire developed for the CCSE survey, an ESRC funded project from 2003–2006 (R000239801). The analysis of this was published in the book *Culture, Class, Distinction* (*CCD*) in July 2009 (Bennett *et al*., 2009). This is basically a replication and actualization, 40 years later and in a different national context, of Pierre Bourdieu’s (1984) original study of *Distinction* in France, which built on the two classic theories of class: Marxist (emphasizing class formation and experience on the basis of the material nature of inequalities derived from ownership of the means of production, identifying two classes) and Weberian (stressing market processes – skills, expertise – for the creation of chances and inequalities, identifying four classes). CCSE also draws upon a similar study in Australia (Bennett *et al*., 1999).

The class analyses of *Distinction* and of *Culture, Class, Distinction*, downplay concerns with property and market relations to emphasize the interconnections and weight of multiple forms of capital. However, it is recognized that property and market relations are embedded in the ways various forms of capital are related to life chances, cultural orientation and political allegiance. The notion of capital mobilizes social power for its operation, as capital is effective when convertible to other resources to be used to gain advantage.

Both *Distinction* and *Culture, Class, Distinction* draw on occupations to identify social classes. They both employ the inductive approach based on Multiple Correspondence Analysis (MCA). In *Distinction* the interplay of economic, social and cultural capitals is plotted independently of socio-demographics. In *Culture, Class, Distinction* cultural tastes and participation are mapped, with supplementary variables also being plotted independently. The MCA is a sophisticated model for analysis of social dynamics and it presents ‘simultaneous complications in several dimensions’ (cf. Saussure in Bourdieu, 1984: 126). Once the various levels and types of engagement of individuals with the cultural items selected in the investigation are plotted and the capitals’ configuration is established, occupation and other variables like gender, age, level of education and income are distributed alongside the axes of the capital distribution of individuals in social space, as supplementary variables. Clearly, this MCA social space is shaped by class divisions, and it also shapes divisions of class, the latter being a clear conceptual difference from the assumptions of the Goldthorpe model (1980). In Goldthorpe, the social space is a given, based on distinctions derived from a service relation or a labour contract one.

For Bourdieu in *Distinction*, the organization of social space is related exclusively to class: derived from the plotting of occupations (a very ‘pertinent’ criteria in Bourdieu, 1984, modelled on the French census) over the distribution of volume and composition of cultural and economic capital.

For Bennett and colleagues in *CCD*, the imprints of class are compounded by other variables. But the study affirms that ‘the most powerful dimension of cultural difference … reflects what Bourdieu called “total volume of capital”, holdings of cultural and economic assets, which form the basis of social class structure’ (Bennett *et al*., 2009: 251). Chiefly, class has an occupational and an educational gradient, which is more salient in some fields than in others. These two indicators *–* occupation and education – correlate strongly because the distribution of economic capital (the key asset for class position) forms the basis for the distribution of the other forms of capital: cultural and social. The *CCD*’s biggest contention with Bourdieu’s *Distinction* concerns the role of social class. In *CCD* patterns of British class behaviour are not as unified and highly integrated as in the French *Distinction*. Individual engagements and engagements of groups do vary within classes, which work as force fields. There is a large degree of overlapping of class tastes indicating that many activities are common to all, that boundaries of taste are permeable across classes and fields, that ‘…some members of the working class share more or less exactly their tastes with some members of the professional-executive class’ (Bennett *et al*., 2009: 252), though this does not eliminate distinction.

Crucial in the explorations of cultural assets in class formation, carried out in *Distinction* and in *CCD*, is the attention to an additional dimension of the economic concerns with property and market relations: the ways in which culture operates to reproduce and reinforce distinctions.

*CCD* claims to be ‘the most sophisticated study of cultural capital ever conducted in Britain’, which subsequent assessments of the work have so far confirmed (eg Weininger, 2010; Gibson, 2010; Petev, 2010; Duval, 2010; Ulas, 2011; Frank, 2012; Coulangeon and Duval, 2013). The team was constituted of four co-investigators for the ESRC grant proposed in 2002 by Tony Bennett (principal applicant), Mike Savage, Elizabeth Silva and Alan Warde. Two research assistants joined the project: David Wright and Modesto Gayo-Cal. All researchers published a number of papers co-authored or single-authored and a major jointly authored book in July 2009 (Bennett *et al*., 2009). The project was also fortunate to secure the collaboration of Henri Rouanet and Brigit Le Roux, who had worked with Bourdieu on *Distinction*. Many other researchers contributed to CCSE’s development as a multi-method research study (see acknowledgements in *CCD*).

There are some parallels between *CCD* and GBCS. Time-wise they are extremely close, the theoretical frameworks are broadly the same, and the population concerned is the same national formation. In the April GBCS 2013 paper it is said that *CCD* offered an empirical platform to create the GBCS new model as far as cultural capital is concerned. In the 2014 GBCS paper *CCD* is invoked more centrally. Savage and colleagues clarify some of the relationship between their 2013 paper and previous work, paying scholarship debts ‘especially that in *Culture, Class, Distinction*’, but no elaboration about connections has been developed.

Other comparisons between GBCS and *CCD* identify that both go beyond the NS-SEC’s model of class; both inductively classify social position based on particular measures of economic, cultural, and social capital; both identify level of education as highly important in social position, with demographic factors such as age – the highest marker of taste – and gender, as well as ethnicity, as relevant factors affecting measures of capital. The profiles of individuals surveyed that have been found to illustrate GBCS classes (Savage, 2013 – power point presentation at Manchester University) are exactly the sort of work that *CCD* researchers innovatively developed (see Bennett *et al*., 2009: ch. 4).

Being a study of cultural capital, *CCD* is *de facto* a study of social class. Although in the April 2013 GBCS paper *CCD*’s work is briefly acknowledged, no reference is made to those *CCD*’s findings which inform the GBCS class analysis. Yet, as GBCS replicates some of the elements used in *CCD*, attention to the legacy of CCSE-*CCD* would possibly have improved sociological stratification analysis in the new project.

Unfortunately GBCS did not present itself as being critically informed by *CCD*. For instance, there are clearly some matters which *CCD* failed to address as well as needed, as for example the importance and valuation of local working-class culture (and the cultural practices most effective in defining working-class participation), as noted in the book, and the effects of the lifecourse on different cultural engagements of women and men, explored after the publication of the book (see Silva and Le Roux, 2011).^5^

GBCS is presented in April 2013 as a completely ‘new model’ of class analysis. Yet, GBCS does not appear comprehensive in measuring a whole class structure. It is clear that the *CCD* study would not have formulated the seven classes presented in GBCS, on the basis of CCSE data. CCSE-*CCD* had no robust evidence for the development of a completely new ‘class model’, nor did it intend to develop one. GBCS does not seem to have robust evidence for a new class model, though it did intend to develop one.

CCSE-*CCD* identifies an ‘elite’, a ‘professional-executive class’, an ‘intermediate class’ and a ‘working class’. It identifies differences between sections (or fractions) within these classes, like ‘higher professionals’ and ‘lower managers’, or, within the working class: ‘lower supervisory’, ‘semi-routine’ and ‘lower-technician’, showing that these have different levels of participation (engagement) with selected cultural activities, different political views and social contacts. *CCD* classes with class fractions are organized into seven groups. Coincidentally this is the same number as in GBCS’ ‘new model’.

It is important to consider the methodological principles informing both studies. They are broadly similar in terms of the content of the survey questionnaire (though very different in procedures for gathering information: CCSE applied an interviewer-trained face-to-face questionnaire to a stratified, clustered, random sample and GBCS performed a self-selected Internet one) and close regarding the application of the general principles of the Bourdieusian approach to the relations of culture and class relations. They also share in the ways these differ from other approaches like those of Wright (1985) and Goldthorpe (1980). However, the picture of class that emerges from the GBCS is very different from the one appearing (not innovatively) in *CCD*, and it relies on different methods for the survey data analysis – GBCS arrive at seven main classes by using latent class analysis (I explore this below), whereas *CCD* presents three main classes (seven class-fractions) through the analysis of the overlaps between the ellipses of different occupational groups, by means of an MCA. How would the GBCS classes appear had the MCA method used in *CCD* been applied in this aspect of the analysis of the GBCS data? How can it be explained that two surveys using broadly similar data, carried out about seven years apart, and orientated by the same theoretical approach should arrive at varying depictions of class divisions?

For the proper advancement of social science knowledge of stratification it would be most worthwhile to reflect upon what in *CCD* was not helpful to GBCS. What did CCSE-*CCD* do to spark off the inauguration of a new phase of class analysis so soon after it was carried out? On the basis of the account of theoretical or empirical engagement of GBCS with CCSE, the work presented in *CCD* did very little. GBCS emerges from different contexts: those of the BBC large online survey and public impact.

## The (big) data

The notion of big data in sociology dates from 2011 (Burrows and Savage, 2014). Big data has been defined as ‘vast amounts of data’ in the *Big Data* journal overview (http://www.liebertpub.com/overview/big-data/611/), while the journal *Big Data & Society* explicitly does not settle on a definition, but defines its remit as that related to a ‘high volume of data’ generated via digital infrastructures (http://bigdatasoc.blogspot.co.uk/p/big-data-and-society.html). Whatever the definition, ‘GBCS is Big Data only in relative terms’ (Burrows and Savage, 2014): it has an unusually large sample of a self-applied ‘fairly conventional’ Internet social survey.

An important reference concerning the development of the GBCS with the BBC project is the claim that it provided a unique opportunity for academic investigation. The researchers state that as the BBC approached them to undertake a study of class, this was an invitation that could not be refused. The BBC project offered the possibility of sociological engagement with digital data, which offered access to a very large sample size. In the 2013 paper it is claimed that this is needed to unravel interactions between the three types of capital. It is stated in the papers of 2013 and 2014 that the web survey offered the ability to ask comprehensive questions in a survey.

The need for large data sets for certain matters to be comprehensively addressed and to gather robust answers has been debated in academia, in particular with reference to the increasing recent digital generation of information. What is relevant in the claim of the GBCS team is that the production of a large data set to study social class is very expensive by academic standards but not by those of the BBC. And big data can be quickly generated only if infrastructural apparatus is available. On the other hand, knowledge produced by other qualitative means, ethnographically, for instance, takes a very long time and dedication to produce; and is much cheaper.

To elaborate a comprehensive model of stratification, researchers deal with social issues that may demand to be statistically measured and require quantitative surveys. The cost is one of the reasons why usually large occupational classifications are dealt with by national statistical offices, as they were with the NS-SEC. The GBCS had generated 161,400 respondents to its web survey by July 2011, the sample used for the 2013 paper, and a total of 325,000 respondents between January 2011 and June 2013 (Burrows and Savage, 2014). In the matter of numbers surveyed, the BBC offered an unequal quantitative opportunity. According to the researchers, it offered an attractive possibility of engagement with digital data.

But, what sort of data resulted from the Internet survey? Not exactly what was needed to measure what was intended. The survey was based on those who could spare 20 minutes on a BBC web survey. This revealed a self-selecting large concentration of the well-educated, in high occupations. There was a strong professional middle-class bias.^6^ (See Savage, 2015, and Devine and Snee, 2015.)

A lot of excellent research produces outstanding findings with small samples, or on the basis of case studies. An example is Skeggs’ (1997) *Formations of Class and Gender*, based on an ethnographic study involving 174 women. Another example is Devine’s (2004) *Class Practices* with about 200 interviews in the UK and US. Yet another study is Savage *et al*.’s (2001) about the northern English middle class. But qualitative cases address different matters.

Despite the GBCS not dealing with big data, some of the discussion about big data is applicable to the large Internet survey of GBCS, one major issue concerning their production designed for ends not directly suitable to particular kinds of analyses. In a recent paper, Kitchin (2014) argues that big data can ‘enhance the suite of data available for analysis and enable new approaches and techniques, but will not fully replace traditional small data studies’. He adds that the resilience of small studies is due to the fact that ‘it is unlikely that suitable big data will be produced that can be utilised to answer particular questions’. This was the case with the GBCS, though not only regarding particular questions but in reference to the very major question of the study: the population distribution of the BBC web survey was statistically limited for the class distribution of the UK population, as measured by NS-SEC, or CCSE in *CCD*.

As a result of this acknowledged limitation, the team took the decision to develop a face-to-face survey with identical questions to a quota sample of 1,026 respondents in the UK, carried out by GfK in April 2011. The issue of it being a quota sample has been discussed and acknowledged. Mills (2013) noted that the original paper has no information about the target sample, the quota controls applied, their interlocking, the application and provenance of weights and their mode of administration; there is no room to infer about uncertainties (see also Mills, this volume). The model does not allow for refined analysis. But the research team claimed they were limited by what it was possible to have.

To strengthen their limited sample, the use of the GfK was aimed at enhancing the data. GfK is an advertising/consumer research company providing market insight in product design and branding. Its focus is on automotive, consumer goods, energy, fashion, financial services, media and entertainment. Supposedly the BBC as client fits under the latter label. According to the website, GfK operates in over 100 markets every day. Does it matter what GfK is?

In social sciences thinking about ‘The Social Life of Methods’ (SLOM), strongly branded within CRESC,^7^ it is recognized that the ways in which data are generated bear on the sorts of knowledge created about the social, or, even more strongly, on how the social is constituted. These ideas borrow from Feminist Studies and Science and Technology Studies (Haraway, 1985; Harding, 1986; Hartsock, 1998) to stress that methods are not innocent and they help to shape social practices. As methods help to create knowledge and the social, how do corporate and private interests like those of the BBC and GfK intervene in the classification of UK social class structure? Clearly the exploration of this goes beyond an account of procedures and inclinations of the various agents involved, although this account helps to clarify allegiances and some implications of the knowledge created (see Devine and Snee, 2015).

To take the similar case of the CCSE survey: researchers went through an Open University procurement process and chose not the cheapest, but the most reputable survey agency for the terms of the investigation envisaged. The survey was carried out with the National Centre for Social Research (NatCen). The grant holders carried out the training of interviewers, developed the coding and oversaw the analysis.

We know nothing about how GfK developed the survey, but the manner in which data was generated may have implications. This reflection relates to a big question about the sociological use of big data (or large online data) generated by current digital possibilities. The GBCS experiment illustrates significant problems.

The representative GfK survey is very small. This is a point criticized by other sociologists (see chiefly Mills, 2013, 2014, 2015; Lambert and Griffiths, 2013). Yet, without the GfK data the large GBCS BBC web sample is ‘useless’ (Mills, 2014: 439). The team sought to expand it by linking results with details on the GBCS. But there is no indication about how the detail of the BBC web survey has assisted understanding of GfK data. To an unreliable sample of 161,400 digital survey respondents another weak small quota sample was linked.

The most problematic matters concern the relationship between the class profiles’ distribution in the GfK survey and the BBC web survey, for this is the basis for the claim to the development of a new class model. First, how can the 2 per cent ‘traditional working class’ group of the GBCS BBC web survey illuminate with ‘fine details’ (presented as the team’s intention) the numerical representation of 14 per cent of this group in the GfK survey? Secondly, how can less than 1 per cent of ‘precariat’, which appeared in the GBCS web survey, substantiate detailed knowledge about the 15 per cent ‘precariat’ in the GfK survey? The numerical difference here is too large, as if these constituted disparate groups; yet, they are reported as supposedly the same in the investigation. Thirdly, surely in these cases (and here the class of the ‘new affluent workers’ is another group example) where proportions from the GBCS BBC web survey are very small, the fine-grained details are poorly substantiated. Three out of GBCS’s seven classes have this problem.

In the face of these issues, it seems worth asking – and this is a provocative question – whether the team needed to do latent, inductive, class analysis to get to GBCS’s seven classes. Given the current knowledge available – consider here CCSE *CCD*, various studies by Savage (and with collaborators), Atkinson (2010), Grusky and Weeden (2008), Oesch (2006), all of which are referred to in the April 2013 paper – would it not be the case that these newly created categories could be made sense of without this kind of data? What are the data doing for the thesis that a new class model was needed and what are the new data revealing of new class formation, class experiences and dynamics of class relations in the UK? What in the BBC web survey data makes the case for the creation of the seven classes credible and a new phenomenon? The increased polarization between the top and bottom is not a new discovery. In what reliable new ways do the GBCS data reveal the fragmentation of the middle of the class structure?

In the GBCS latent class analysis was used to identify where the class boundaries lie. This was applied to the GfK survey, with the explication of the boundaries relying on the BBC web survey data. Both the clustering that formed the classes and the socio-demographic variables correlated to them are GfK data. This material is then claimed to be enhanced by the ‘granular detail’ of the BBC web survey regarding information on specific jobs and geographical location. A close look at the data raises concern about the reliability of this transposition. For instance, in the elite group classification, which in the GBCS BBC web survey amounts to 21.8 per cent of respondents (a large group for an elite, indeed), there are 60.5 per cent chief executive officers and 44.4 per cent dental practitioners (selected among ‘the most “over-represented” occupations’ in the elite class). While these were not placed as ‘elite’ purely on the basis of their occupational label, and the model predicates that ‘there is no clear affiliation between specific occupation and a latent class’ (Savage *et al*., 2013: Table 8 and p. 245), the criteria and procedures for the classification are not made clear and do not appear convincing, as far as the proportions of occupations grouped within classes are concerned.

I asked above how it can be explained that two surveys – the CCSE and GBCS – using some similar data, time-wise close, and orientated by the same theoretical approach should arrive at varying depictions of class divisions. It is possible that the simplified GfK survey, with the dispersed and unrepresentative sample of the BBC web survey, did not allow for a fuller MCA exploration; which indicates the limits of the data for the theoretical engagement envisaged.

Questions like these have been put to the team since April 2013 by a number of academics and the researchers have engaged with the debate in a variety of ways.^8^ Relaying to the present three main areas can be highlighted.

First, the academic community has been attracted to engage extensively with the GBCS (it includes this special issue) given the media exposure, the reputation of social science research now fuelled by the London School of Economics’ social media platforms, and the continuing reiteration of its valuable assets by the members of the team. The matter remains in sociology’s public outlets. Savage has been prominent in this. In a recent paper (Burrows and Savage, 2014) he argues that the GBCS stokes tensions between the ‘primacy of orthodox sociological repertoires’ and the new methods eliciting online information and crowdsourcing, hence making a claim that their data are ‘big’. While true that references to these matters are unveiled, the central point of dissent is about the quality of the study, design, data and analysis. Robust dissecting of reservations about the study have been presented, as well as (more diffuse) scepticism and ruminations, but no defence or embracing by other academics have been presented, except perhaps by the *Sociology* editors, understandably pleased with the exceptional number of downloads (Woodward *et al*., 2014). The GBCS remains highly contentious among more and less orthodox analyses of class because method, quantity and quality have been scrutinized, but also due to the ability to theoretically extrapolate findings from the contradictory data between the BBC GBCS and the consumer research company, GfK.^9^

Secondly, the claim to the development of a ‘new model of class analysis’ appears to have been dropped; or it is now downplayed. An interesting turn now stresses the need to consider ‘the very real limitations of relying on sources such as the GBCS for comprehensive accounts of class relations’ (Burrows and Savage, 2014). This softens considerably the earlier claims in the original paper. This is welcome.

Thirdly, the analytical focus has now been restricted to claims about the elites, defined as the ‘new corporate managerial group’ (see Savage, 2015), measured in the web survey as representing 21.8 per cent of the population. This is also welcome. I commented earlier that the upper strata considered is wider than the usual sociological classification of elites, as understood in CCSE *CCD*, for example, where it sits above the professional-executive group, but they represent the powerful and privileged at the top. In this regard, it is possible that GBCS is correcting or improving on *CCD*, though there is not yet any argument about this.

## Public interest and impact

In the current wave of big data production, the academic authority to define social knowledge has been challenged, demanding critical evaluations of authority and the role of academics. Considerable appropriations of what qualifies as the social, and understandings about its operations, have been claimed by the public. This is one of the very outcomes of social sciences’ success (Sewell, 2005). Most people are acquainted with sociological categorizations, jargons and meanings; reflections about the workings of patterns in social life spread widely and have become part of everyday conversations.

However, the laudable fact that people learn about social matters and are empowered by this, like patients who learn about ills and cures, challenges the craft of the sociologist, or the medic. The engagement with non-academic audiences is necessary but fraught with risks, for example to do with the potential entrapments of the media, the ignorance of social scientists about the possible pitfalls, and the implications of inevitable misinterpretations of circulated knowledge. Michael Burawoy’s 2004 presidential address to the American Sociological Association, ‘For Public Sociology’, refers to some issues relevant to a critical review of this case. His claims for public sociology articulate the need for autonomy of discovery by researchers in a way that engages ‘multiple publics in multiple ways’, while remaining relevant and engaged to public issues and debates (Burawoy, 2005). That people learn that sociology creates robust knowledge about what matters in everyday life and in the invisible patterning of social advantages, is to be applauded. This is claimed as the intention with the GBCS experiment.

What happened? In brief: the GBCS created a lot of headlines, a huge volume of tweets, great presence on Facebook, nearly 7 million visits to the BBC site, it was talked about on radio, television, nationally, in countries beyond the UK and it provoked major waves, as illustrated in Devine and Snee’s paper (2015). They argue that the significance of talking to a national audience about Bourdieusian-influenced class analysis should not be underestimated. The GBCS reproduced again through its experiment the British obsession with class.

The evaluations of the good the experiment delivered for the British public interest and for British sociology raise vast questions and contentious positions. While it can be said to be excellent to have the subject of social class featured widely in the media, much of the manner in which this was done presented sociologists as somewhat churning the obvious with no scientific rigour, and left room to ridicule the discipline.^10^ The exposure put enormous pressure on the research team. At the 2013 CRESC Annual Conference Mark Taylor, from the research team, said: ‘it was like getting hundreds of reviewers’ comments in one go’. Could this pressure have been helped? What measures would have alleviated the exposure or controlled the content? Does it matter at all that things happened the way they did?

I reflect upon these issues of the public relations aspect of the study – relations with the media, researchers’ engagement ability, and misinterpretations of knowledge – by reference to the content of dissemination and the academic reputation of researchers. These are linked to discussion about the values of the impact of knowledge and the case of public interest.

I am mindful that the academic content needs to be distinguished from the media audience one but they are too closely linked in the case of the GBCS. A key academic contention was and is about deep divisions in the understanding of social stratification as a theoretical and empirical matter. It is, however, also, and perhaps above all, about the quality of the empirical exploration, which is masked by the claim concerning the devaluing of the theoretical underpinnings.

I addressed some quality issues above. Some of these are related to the relationships between the team/investigation and the media/BBC. There are no media scholars among the GBCS team. The academy and the media are very different fields, sometimes with opposite interests. Academics need to be savvy of the operations of the media to effectively negotiate the terms of engagement. One potential problem is illustrated by the use of the Class Calculator, something that happened later in the process, but was at the heart of the exposure. It simplified the longer survey questionnaire to be answered in one minute as a quiz to locate individuals in the class structure. I took this one recently and changing my reply to just one question – preferring rock to hip-hop – I was positioned into the elite instead of among the established middle class. Many comments in this regard were presented in social media in April 2013 with accompanying jokes (see note 10). The Class Calculator was a quiz to play with. It was fun because, like talking about soaps, as a culture we enjoy things we can share with others and gossip, or join in; and matters of moral reasoning are great social glues. But unless social scientists properly study these phenomena as media productions, drawing on a range of academic media expertise – the ways people engage with these fun matters – not much can be said about patterns, relations and links. The same goes for enacting social research methods as, in order to ‘curate sociology’ (cf. Puwar and Sharma, 2012), researchers take various thoughtful measures about process and analysis. The GBCS performed sociality, but not in a methodical way involved in a process of enacting classifications of social class. The enactment process was haphazard. Because of this it became difficult to respond to the media exposure in a controlled manner, and it was not possible to identify robust evidence for the classification presented. Misinterpretations are always possible, but easier to correct with validated data.

Perhaps a lesson in caution from this part of the experiment involves the valuation of data as scientific resource, whereby conditions for dissemination become supported and regulated according to impact. This BBC GBCS had huge impact. But the valuation of research needs to focus beyond the numerical, on advancement of knowledge and social relevance of matters on quality not quantity. Apparently the GBCS team is engaged in an ongoing study of the reception of its findings and this reflection is to be welcomed. Attention to the effects on research of current drives in universities’ responses, with market solutions, to the intensified competition for resources, is a particular pertinent issue.

This bears on relations of academic consecration and on what counts as reliable knowledge. Burawoy (2005) warned against the temptations of academics to compromise professional and critical commitments in pursuit of popularity and intellectual vanguardism. Academics occupying low and high positions in the academic field learn the implications of their practices in relation to the positions they occupy: junior positions are not conducive to large or strong interventions, and juniors in research teams may find themselves unable to diverge. Since the GBCS team claims an asset of the project was the dissemination of Bourdieu’s ideas, another important point of discussion is illustrated by Bourdieu’s account of how academic reputation allows one to dare to run against orthodoxy:

Saying [in *The Weight of the World*] that an interview was a spiritual exercise was hard. I have always thought that, I have always felt it. But there was this kind of positivist repression (*refoulement positiviste*): a questionnaire must be scientific (*rigoureux*), objective, neutral, there’s no cathexis (*investissement*)… You must also have known this form of masochism, which passes for professional virtue. I had to wait to be the age I am now, and to have the social guts (*culot social*) that come with it, to be able to transgress like that. (Maître and Bourdieu, 1994: xiv–xviii, in Darmon, 2015 [her translation])

By referring to his current ‘social guts’, Bourdieu stated not only his age but, above all, his consecrated position in the field of sociology in 1994, which had allowed him to break with old habits, both individual and collective, regarding what was deemed scientifically proper in a field inherited from 19th-century French sociology. But this carries great responsibility, without which reputations cannot be sustained. Institutional trappings of scholarly positions are accompanied by requirements of representing acts and ideas within the web of collective expectation and obligation in a discipline’s field, and the politics of particular institutions in this field.

The current productivity-performance led impact climate in British academy appears to have been consequential for the emergence and handling of GBCS, as citation metrics, media presence and general engagement of publics have become highly valued. Discussing the difference that quantity makes, Leonelli (2014) disputes the claim of comprehensiveness allowed by big data regarding this as potentially misleading rather than helpful in shaping public perception of research findings. The practice of big data production reinforces the emphasis on products, disregarding the process whereby results are achieved. This has been a central point of contention in the academic debate about the GBCS: how data was generated, measured, analysed. The tendency fits in with the ‘prominence attributed to data as commodities with high scientific, economic, political and social value’ (Leonelli, 2013, cf. 2014).

These reflections also resonate with Sabaratnam and Kirby’s (2014) arguments that big data and judgements of what good academic research is, have been entangled in recent uses of metrics in research assessment. Apparently this has not yet fully reached social sciences (except for economics), but its application is a warning. Work can be cited because it is good (well known, correct and offers a good case), and for not good reasons (it is incorrect, we disagree, it says something outrageous, provocative, unhelpful and misleading), as well as for neutral reasons (it links to one’s work, was published in a particular journal). Academic fame is significant to the game as they note that the system privileges higher academic positions, discriminating against women, ethnic minorities and less-established scholars. It may entail patronage exchanges with junior academics.

These matters also affect journal editors’ decisions concerning the impact factors of their publications, as well as commercial publishers and learned societies. The April 2013 paper went through the refereeing process very quickly and was published for an early online availability to coincide with the BSA annual conference. It gave great publicity to all. The debate that ensued carried on filling the pages of UK sociology journals. Now, with things better settled, it appears valuable to expand reflection upon developments and findings of the GBCS into the development of the academic agenda for the incorporation of culture into social stratification analyses, and also for the effects of the impact agenda and big data on social science discoveries and the proper engagement of publics. Public value and economic value can be confused in the agendas of funders. It is worthwhile to ask: how was GBCS data shaped by the tools that created them? Tools always shape results. How can this data be valuable for the BBC? How does the experiment shape sociology in the process of the ‘ongoing historical invention’ (cf. Wacquant, 2013: 22) of the discipline? I hope to have made here some small points towards addressing these questions, so we can learn more about the GBCS as a limit case in thinking about neoliberal research drives of impact, big data, and the privatization and marketization of knowledge production. Critical engagement with the GBCS is important to help inform future research in sociology, in particular research that involves collaboration with media, public bodies and commercial entities.

## Notes

1 See Flemmen (2013) for fine reflections about this.

2 The first sentence in the GBCS web survey presentation on the BBC site reads: ‘It has often been said that the British are obsessed with class.’

3 See blogs.lse.ac.uk/impactofsocialsciences/2013/04/23/the-making-of-the-great-british-class-survey

4 It is not entirely clear what these phases are. Following the official national classification, the first phase would be the one supplanted by the NS-SEC in April 2001. Prior to this, a combined classification from the Registrar-General’s Social Class (RGSC), originated in 1913, and Socio-economic Groups (SEG), originated in 1951, privileged skills. The intellectual origin of the NS-SEC is the John Goldthorpe schema (otherwise known as the Nuffield Class Schema developed in the 1970s). It differentiates the employment relation as either service relationship or labour contract on the basis of three criteria: form of remuneration, career opportunities and time autonomy. The NS-SEC emerged from the ESRC review of government classifications undertaken between 1994 and 2000. For an excellent account of the history of socio-economic classifications in the UK and the development of the NS-SEC see Rose and Pevalin, 2001. (In the original GBCS paper, Savage *et al*., 2013: 201, the first phase is said to have lasted to the 1980s, the second phase to have been initiated from the 1970s).

5 Another missed opportunity of the GBCS to improve on the CCSE refers to the indicators used to measure social capital. In the GBCS questionnaire a list of 37 occupations is offered with the question – what sorts of people you know – asking if these are known ‘socially’ and how many are known. In the CCSE only 11 selected occupations are listed (representing the class/fractions groups) and the kind (not the quantity) of social contact is probed: a member of my close family, another relative, a friend, an acquaintance, a neighbour, someone I know from work or study, other (write in). These more refined CCSE categories are made invisible under the broad category of knowing ‘socially’ used in the GBCS.

6 Danny Dorling (11 April 2013) said: ‘The most interesting finding from the academic article was that the Elite are more than 50 times likely to fill in the online BBC survey than the bottom group the academics called the “precariat”’ (www.statisticsviews.com/details/feature/4582421/What-Class-Are-You.html).

7 The Centre for Research on Social Cultural Change (CRESC) developed from 2009 the cross-cutting theme of the Social Life of Methods (SLOM) focused on the implications of situated knowledge practices on knowledge content.

8 I had occasion to do this publicly as invited discussant of a sub-plenary on the GBCS at the Annual CRESC conference in September 2013.

9 Savage *et al*. (2014) recognize that the academic reception of their study has been ‘highly critical’, registering hostility and scepticism.

10 See a thoughtful discussion by David Hill (Liverpool University) ‘Precariousness and Public Sociology’ in http://schizomedia.wordpr3ess.com/2–13/04/08/precariousness-and-public-sociology/

## Notes

Please quote the article DOI when citing SR content, including monographs. Article DOIs and “How to Cite” information can be found alongside the online version of each article within Wiley Online Library. All articles published within the SR (including monograph content) are included within the ISI Journal Citation Reports® Social Science Citation Index.
